# Lysosome-associated CASM: from upstream triggers to downstream effector mechanisms

**DOI:** 10.3389/fcell.2025.1559125

**Published:** 2025-03-27

**Authors:** Namrita Kaur, Sven R. Carlsson, Alf Håkon Lystad

**Affiliations:** ^1^ Centre for Cancer Cell Reprogramming, Faculty of Medicine, University of Oslo, Oslo, Norway; ^2^ Department of Molecular Cell Biology, Institute for Cancer Research, Oslo University Hospital, Oslo, Norway; ^3^ Department of Medical Biochemistry and Biophysics, University of Umeå, Umeå, Sweden

**Keywords:** CASM, VAIL, STIL, autophagy, lysosome damage, atg8ylation, Atg8

## Abstract

Lysosomes are dynamic organelles critical for cellular degradation and signaling, safeguarded by a limiting membrane that prevents leakage of harmful contents into the cytoplasm. Upon lysosomal damage, cells deploy defensive mechanisms, including a key process called CASM (conjugation of ATG8 to single membranes), which lipidates ATG8 proteins onto the limiting membrane to support protective pathways. CASM operates through two pathways: VAIL, induced by lysosomal pH changes via V-ATPase and ATG16L1, and STIL, triggered by sphingomyelin exposure and mediated by TECPR1. This review examines CASM’s role in lysosomal damage responses, exploring the mechanisms of damaging agents, distinctions between VAIL and STIL, and the downstream effects of decorating lysosomes with ATG8, including effector recruitment for membrane repair or removal.

## 1 Introduction

The degradative system that resides inside lysosomes is highly effective, comprising a large array of highly active enzymes in an acidic environment (pH 4-5) that is capable of degrading most of the incoming material (De Duve and Wattiaux, 1966; Ballabio and Bonifacino, 2020; Settembre and Perera, 2024). This destructive capacity underscores the need for a robust and tightly regulated cellular response to lysosomal damage, ensuring that harmful contents do not leak into the cytoplasm and compromise cell viability. Recent studies have uncovered a range of protective mechanisms that either restore or eliminate damaged lysosomes, including membrane repair pathways and targeted degradation processes (Papadopoulos et al., 2020; Zhen et al., 2021; Zoncu and Perera, 2022; Duran et al., 2024). These mechanisms rely on sophisticated protein machinery, including the ATG8 conjugation system, first characterized in autophagy, a conserved pathway for cellular recycling and homeostasis (Mizushima et al., 2011; Yim and Mizushima, 2020; Durgan and Florey, 2022; Figueras-Novoa et al., 2024). In response to lysosomal damage, ATG8 proteins and their conjugation machinery function in two distinct pathways: autophagy and CASM. While we will briefly discuss both processes, this review primarily focuses on CASM and its established roles at compromised lysosomes.

### 1.1 Autophagy and selective autophagy

The principal mechanisms of canonical autophagy (often referred to as macroautophagy) are now well-established, including an initiation phase near the ER membrane, the growth of a double-membraned phagophore/isolation membrane, followed by capture of cargo to be degraded as bulk, or through specific adaptors/receptors in a process called selective autophagy (Yamamoto et al., 2023). Closed phagophores, called autophagosomes, with its captured cargo (which can be whole organelles tagged for destruction) then fuse with functional lysosomes forming autolysosomes in which the degradation takes place through the action of lysosomal enzymes.

The selective autophagic process responsible for removing non-functional damaged lysosomes is called macrolysophagy (Maejima et al., 2013; Gatica et al., 2018; Vargas et al., 2023). The recognition of such lysosomes is thought to be mediated through the exposure of internal structures (caused by disruption of its limiting membrane), which are recognized by lectin-type proteins present in the cytosol belonging to the galectin family (Johannes et al., 2018; Jacob and Gorek, 2024). The galectin reaction leads to ubiquitination of the lysosome that is recognized by autophagy receptors, which function to cross-link the damaged lysosome with the phagophore membrane, facilitating sequestration and delivery to a healthy lysosome (Lamark and Johansen, 2021).

### 1.2 Atg8ylation

Generally, the role of receptors in selective autophagy depends on the lipid-conjugation of mammalian Atg8 homologs—here referred to as ATG8 — which decorate the surface of the phagophore. These receptors typically bind to ATG8 through specific sequences known as LIR (LC3-interacting region) motifs (Johansen and Lamark, 2020). ATG8 proteins have been exploited during evolution and come in slightly different versions and in varying numbers in different species (Mizushima, 2020). In humans, six alternative homologs are expressed: LC3A, LC3B, LC3C, GABARAP, GABARAPL1, and GABARAPL2. Additionally, a seventh variant designated LC3B2 is described (Shpilka et al., 2011), distinct from LC3B by just one amino acid. Although they have been extensively studied for many years, their differential functions are still not entirely clear (Rogov et al., 2023). However, phylogenetic analyses have shown that members of the GABARAP subfamily are more ancient in evolution and appear to have more prominent roles in specific reactions such as receptor binding and generation of functional phagophores (Johansen and Lamark, 2020; Mizushima, 2020).

**FIGURE 1:**
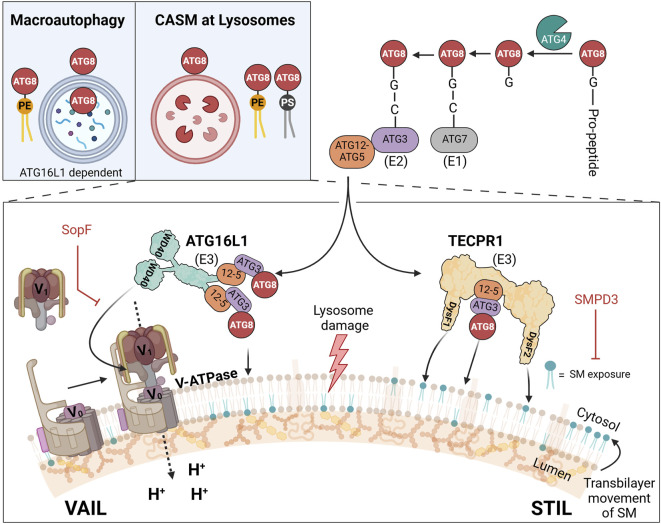
CASM at lysosomes: distinct VAIL and STIL axes in ATG8 lipidation upon membrane damage. Upper left inset: comparison of double-membrane atg8ylation in macroautophagy and single-membrane atg8ylation in CASM at lysosomes. In macroautophagy (left), ATG8 proteins are lipidated by phosphatidylethanolamine (PE, yellow) on double-membraned phagophores, which mature into autophagosomes. In CASM at lysosomes (right), ATG8 proteins are lipidated by PE or phosphatidylserine (PS, black) directly on the single membrane of lysosomes in response to damage. Illustration of the two known CASM axes that respond to lysosomal damage: the VAIL axis (left) and the STIL axis (right). In the VAIL axis, dissipation of the proton gradient triggers assembly of the V-ATPase complex, which consists of the cytosolic V_1_ subunits and the membrane-integral V_0_ subunits. This assembly creates a conformational state recognized by ATG16L1 via its WD40 domain, facilitating ATG8 lipidation on de-acidified lysosomes. In the STIL axis, sphingomyelin (SM) exposure on the cytosolic leaflet of the lysosomal membrane recruits TECPR1 through its DysF domains, also leading to ATG8 lipidation. The ATG12-ATG5 (“**12-5**”) conjugate interact with ATG16L1 (VAIL) or TECPR1 (STIL) to form E3-like complexes. ATG8 proteins are processed by ATG4, activated by ATG7 (E1-like enzyme), transferred to ATG3 (E2-like enzyme), and conjugated to PE or PS on the membrane via either E3 complex.

The lipid conjugation reaction of ATG8 proteins is a dynamic multi-factorial process (Mizushima et al., 2011; Ohsumi, 2014; Lystad et al., 2019) (Figure 1). The last reaction step is the transfer of activated ATG8 from ATG3 to aminophospholipids (phosphatidylethanolamine, PE, or phosphatidylserine, PS) in the receiving membrane (Durgan et al., 2021). This reaction is enhanced by the E3-like ligase ATG16L1, which, together with other factors, determines the specific target membrane within the cell (Fujita et al., 2008). Collectively, this process of covalently attaching ATG8 to membrane lipids, specifically PE or PS, is referred to as “membrane atg8ylation” or “ATG8 lipidation” (Deretic and Lazarou, 2022). While atg8ylation is reported to also occur on proteins (Agrotis et al., 2019; Nguyen et al., 2021), this review focuses exclusively on its lipidation. Therefore, we hereafter use “atg8ylation” specifically to denote the lipidation of ATG8 proteins.

### 1.3 CASM

Classically, the atg8ylation reaction proceeds on the autophagic membrane, forming the foundation for the functional phagophore. In recent years, atg8ylation has been found to also occur on non-autophagic membranes (Durgan and Florey, 2022), where ATG8 proteins have roles separate from those at the phagophores. Such processes were formerly called “non-canonical autophagy” to distinguish them from canonical autophagy but was renamed as CASM (conjugation of ATG8 to single membranes) which better denote the cellular structures where they appear and further discriminate them from autophagy (Durgan et al., 2021). The designation CASM is now used as an umbrella term for atg8ylations that appear on single-membrane structures at various locations in the cell (Durgan and Florey, 2022; Deretic, 2024; Figueras-Novoa et al., 2024; Kaur et al., 2024).

The lysosome appears to be particularly susceptible to CASM, especially in response to damage, but can also occur on other structures such as Golgi (Gao et al., 2016), ER (Sun et al., 2023), endosomes (Heckmann et al., 2019), phagosomes (Sanjuan et al., 2007), and lipid droplets (Omrane et al., 2023) (note: in the latter case atg8ylation occurs on a single-layer of phospholipids, not a bilayer). The reactions leading to the lipidation of ATG8 differ from those in canonical autophagy, such that factors important in the initiation phase of canonical autophagy (ULK kinase complex, class III PI3kinase complex, and WIPI2) are not needed in CASM (Fletcher et al., 2018; Lystad et al., 2019). Furthermore, although several factors are the same in the later steps of the conjugation reaction (ATG3, ATG5, ATG7, ATG10, and ATG12), there are important differences in the E3-like reaction that are related to its site of action. While canonical autophagy can proceed with only the amino-terminal part of ATG16L1 (which in size and function corresponds to the yeast ortholog Atg16), CASM is strictly dependent on a carboxyl-terminal WD40 domain for binding to the endolysosomal surface (Fletcher et al., 2018; Lystad et al., 2019; Rai et al., 2019). Under specific conditions, the WD40 domain of ATG16L1 engages the proton transporter V-ATPase (vacuolar ATPase), initiating a pathway known as VAIL (V-ATPase-ATG16L1-Induced LC3-lipidation) (Fischer et al., 2020) (Figure 1).

Complementing this, we and others have recently identified a second type of E3-like ligase, TECPR1 (tectonin β-propeller repeat-containing protein 1), which activates during endolysosomal damage in response to sphingomyelin (SM) exposure on the lysosomal surface (Boyle et al., 2023; Corkery et al., 2023; Kaur et al., 2023; Wang Y. et al., 2023), forming an alternative axis termed STIL (Sphingomyelin-TECPR1-Induced LC3-lipidation) (Figueras-Novoa et al., 2024). Although the search for additional E3-like ligases in CASM remains an active research area (Deretic, 2024), only the two E3 complexes—those containing ATG16L1 and TECPR1—currently have documented activity in ATG8 lipidation. Accordingly, we limit our discussion here to the well-characterized VAIL and STIL axes of CASM, mediated by ATG16L1 and TECPR1, respectively.

## 2 The lysosome and its limiting membrane

The lysosome is not a discrete static organelle, but continuously goes through dynamic changes by means of fusion and fission reactions (Bright et al., 2016; Ballabio and Bonifacino, 2020; Settembre and Perera, 2024). The result is that the collection of lysosomes in a cell can vary in size, location, pH, and enzyme content (Bohnert and Johnson, 2022). During the late stages of endocytosis multi-vesicular bodies (MVBs) are formed (also referred to as late endosomes), containing newly synthesized hydrolytic enzymes and material to be degraded (Gruenberg, 2020). MVBs can fuse with old lysosomes with the purpose of reusing enzymes and other material. The fused structure is usually referred to as an “endolysosome,” and it is believed that this organelle is the principle degradative compartment having an optimal low pH and the full set of enzymes (Bright et al., 2016; Johnson et al., 2016; Sava et al., 2024). The endolysosome contains in addition to enzymes and cofactors intraluminal vesicles (ILVs) and lamellar membrane sheets, originating from MVB formation, that have important roles in lipid degradation (Breiden and Sandhoff, 2019b) (see below). During degradation and export of products out to the cytosol, the endolysosome change in appearance and matures into a “terminal lysosome,” accompanied by content condensation and an increase in luminal pH to almost neutral at the final stage (Bright et al., 2016). The latter phenomenon was described to be dependent on the assembly regulation of active V-ATPase (Sava et al., 2024). The terminal lysosome can be reused through fusion with a new incoming late endosome. In addition, at certain stages in the maturation process, membrane tubules are formed from the endolysosomal surface that undergo scission, a process that is believed to give rise to nascent lysosomes but can also have other functions and be cell-type specific (Li et al., 2016; Yang and Wang, 2021; Bohnert and Johnson, 2022). Disturbances in this recycling process is thought to be linked to neurological and other degenerative diseases. Due to its complexity, in many experimental setups it is uncertain which stage in lysosome biogenesis that is affected, for example, during drug treatment. Therefore, in this text the use of “lysosome” or “endolysosome” refers to any structure from late endosomes to terminal lysosomes.

### 2.1 Lysosome limiting membrane components

The lysosomal limiting membrane has a unique, but variable, structure in order to accomplish its special roles (Kolter and Sandhoff, 2005; Saftig and Klumperman, 2009). The inner surface is covered with a thick glycocalyx, a carbohydrate-rich layer mainly composed of two highly glycosylated integral membrane glycoproteins LAMP-1 and LAMP-2 (Fukuda, 1991; Eskelinen et al., 2003). Special glycan structures (polylactosaminoglycans) present on LAMP-1 and LAMP-2 make them vastly resistant to degradation by glycosidases and proteases in the lumen (Carlsson et al., 1988; Kundra and Kornfeld, 1999), and the glycocalyx therefore yields an effective protection for the lipid bilayer and for other proteins on or in the membrane. Most likely, it is β-galactosides within this glycocalyx that is recognized by cytosolic galectins to signal for rupture of the membrane and to trigger removal of the lysosome by macrolysophagy (Maejima et al., 2013). Other membrane glycoproteins constituting the glycocalyx in lower amounts are LIMP-1 and LIMP-2 (lysosomal integral membrane proteins) (Fukuda, 1991; Eskelinen et al., 2003).

#### 2.1.1 Lipids

The limiting membrane of lysosomes is highly asymmetric and is, with some important exceptions, essentially a mirror of the asymmetry of the plasma membrane and endosomes (van Meer, 2011). SM is normally found only on the luminal side while PS, PE, and phosphatidylinositol (PI) are mainly on the cytosolic side. Phosphatidylcholine (PC) is distributed in both leaflets whereas free cholesterol is normally low in the lysosomal membrane but may have important roles in the regulation of lysosomal activities (Maxfield and van Meer, 2010; Meng et al., 2020). Apart from these common membrane lipids, the lysosomal membrane contains in addition an atypical lipid, bis[monoacylglycero]phosphate (BMP), also termed lysobisphosphatidic acid (LBPA) (Kolter and Sandhoff, 2005; Gruenberg, 2020). This lipid, which is not detected elsewhere in the cell, is asymmetrically located on the luminal side of the limiting membrane, and also present on lysosomal internal membranes such as ILVs. BMP is believed to play an important role in protection and activation of the performing lipases and other hydrolytic enzymes in the lysosome (Gallala and Sandhoff, 2011). As described later, the disturbance of asymmetry and/or interference of BMP-dependent binding mediated by foreign substances in the lumen has crucial consequences for the response by proteins in the cytosol.

At certain stages during the lifetime of a lysosome, lipid head-groups on the cytosol-facing monolayer are covalently modified to signal for actions required. As mentioned above, atg8ylation is a model of such an alteration, and phosphorylation of PI are other important examples of modifications of the lysosome that are currently being revealed (Posor et al., 2022). Multiple specific lipid kinases can produce a series of PI variants (called phosphoinositides), of which PI(3)P, PI(4)P, PI(3,4)P_2_, PI(3,5)P_2_, and PI(4,5)P_2_ are found to have differential roles in the complex regulation of lysosome function. For instance, PI(4)P is generated through activation and recruitment of PI4K2A to the lysosomal membrane during damage (Radulovic et al., 2022; Tan and Finkel, 2022). PI(3,5)P_2_, synthesized by PIKfyve (Hasegawa et al., 2017), mediates processes such as tubulation during lysosomal reformation and formation of ILVs that degrade lysosomal membrane proteins following damage (Rodgers et al., 2022; Klein et al., 2024).

#### 2.1.2 Proteins

The limiting membrane has in addition to the major glycoproteins described above a large number of membrane proteins in lower amounts, such as transporters of nutrients and ions, signaling complexes, motor protein adaptors, small GTPases, tethering factors, and SNAREs (Ballabio and Bonifacino, 2020). Perhaps the best studied among them is the active membrane-translocator of protons, termed vacuolar ATPase (V-ATPase) (Collins and Forgac, 2020; Freeman et al., 2023). Its action is fundamental for generating the optimal acidic environment for degradation in the lumen, and in addition the protein complex is crucial for signaling to the cytosol that the desired acidity is not reached or lost (Hooper et al., 2022). V-ATPase is a multi-subunit structure that consists of a membrane-integral part (V_0_) and a catalytic cytosolic part (V_1_). Pump activity is achieved when the full quaternary structure is assembled, and when the desired pH is reached V_1_ dissociates from V_0_ and pumping is blocked (Sava et al., 2024).

When damage disrupts the proton gradient—such as from proton leakage through the membrane—the V-ATPase subunits reassemble. However, this reassembly occurs in a manner that prevents active proton pumping (Timimi et al., 2024). Instead, a conformational change occurs in the V_1_ domain, which is specifically recognized by ATG16L1. This recognition triggers CASM activation as part of the VAIL response (Timimi et al., 2024). This dynamic assembly and disassembly cycle of the V-ATPase is regulated by additional factors, including the RAVE complex (Jaskolka et al., 2021), as well as mTOR and its associated proteins (Liu and Sabatini, 2020). Importantly, CASM appears to rely not merely on the assembly state of the V-ATPase but also on the unique presence of assembled yet inactive complexes. This suggests a dual regulatory mechanism, wherein both the loss of proton gradients and the resulting inactive V-ATPase state serve as critical cues for CASM activation.

Of the so far identified proteins with roles in induction or as effectors of CASM (apart from V-ATPase) only a few are resident in the lysosomal outer membrane. Mostly based on microscopy analysis, a majority of the participating proteins appear to be recruited to (or detached from) the limiting membrane only during the damage response. The role of several such proteins will be described below under “Downstream effectors of CASM.” However, the recruitment of at least some of the cytosolic proteins that participate in damage signaling depend on integral membrane proteins, such as the binding of mTOR to the amino acid transporter SLC38A9 via Ragulator and Rag GTPases (Rebsamen et al., 2015). Another well-studied integral membrane protein is NPC1, which is the principle transporter of cholesterol to the outside of the lysosomal limiting membrane (Meng et al., 2020). Its role in the response to damage remains to be elucidated, but as cholesterol homeostasis is known to be central for lysosomal function, such as in the regulation of mTOR activity (Davis et al., 2021), a contribution of NPC1 is anticipated also in defense reactions when lysosomes are injured (Kendall and Holian, 2023).

Lysosomal resident integral membrane proteins with evident roles in the defense against injury are the various ion channels with different specificities (Riederer et al., 2023), although their involvement in CASM is not fully elucidated. The major Ca^2+^-channel, TRPML1, responds to damage by translocating Ca^2+^ from the intraluminal store to the cytosol where several factors are activated in a Ca^2+^-dependent manner. Interestingly, CASM can be induced by agonists to TRPML1, and in this case atg8ylation occurs without an increase in lysosomal pH or severe membrane damage (Goodwin et al., 2021). Drug and bacteria-induced Ca^2+^- release was also found to trigger a rapid Ca^2+^-dependent scrambling of the lysosomal membrane lipids and exposure of SM to the cytosol (Niekamp et al., 2022). This effect led to the removal of membrane, which was suggested to occur through invagination based on ceramide production by neutral sphingomyelinase on the cytosolic side of the membrane. It can be anticipated that additional resident lysosomal proteins with functions in the damage response will be discovered in the coming years. For example, the Ca^2+^-dependent scramblase described above is yet to be defined (Niekamp et al., 2022).

While this text discusses various aspects of CASM in lysosomal damage responses, it is important to note that certain atg8ylation related findings—such as LC3-Dependent Extracellular Vesicle Loading and Secretion (LDELS) (Leidal et al., 2020) and retromer-dependent trafficking (Paddar et al., 2025)—are not covered in detail here. For example, LDELS involves ATG8-family protein–driven cargo secretion via extracellular vesicles (EVs) but does not involve compromised lysosomes, and neither VAIL or STIL has yet been linked to this process. Likewise, the retromer complex reinforces lysosomal integrity through trafficking, relying on ATG5 and atg8ylation, however no role for VAIL or STIL was identified in this study either. Although these processes are important, they do not fall within the scope of CASM as presented here.

Having discussed these essential aspects of lysosomal architecture and composition, we next look at how a variety of agents—both external and endogenous—are known to induce lysosomal damage and thereby provoke a CASM response.

## 3 Different types of damage that can induce a CASM response

As cells are exposed to many different types of substances, through uptake by endocytosis or by other means, damage to lysosomes can occur in a variety of ways. Lysosomes are designed to take care of natural macromolecules, degrade them into smaller constituents, such as amino acids, monosaccharides, nucleosides, cholesterol, fatty acids, and glycerol, and then transport them over the limiting membrane to be used at other locations in the cell. Although certain substrates in the lumen can possess difficulties for enzymes or in transport, for example, lipid substrates to overcome the phase problem with water (Breiden and Sandhoff, 2019b), intricate systems have evolved that will ensure that no build-up of indigestible material occurs. If that happens it will inevitably lead to disease, as seen in the large group of lysosomal storage diseases (LSDs) (Platt et al., 2018).

### 3.1 Pore-forming proteins

Much of what has been learnt about responses to lysosomal damage comes from studies with pathogenic infectious microorganisms and viruses (Durgan and Florey, 2022; Wang et al., 2022; Figueras-Novoa et al., 2024). A number of different strategies have been revealed that are used by pathogens to overcome the problems with the harsh conditions in the endolysosomal system. A common theme is the production of proteins that assemble and form ion conducting pores in the membrane, denoted pore-forming toxins (PFTs) (Barisch et al., 2023). The assembly of PFTs in the endolysosomal system leads to neutralization of the lumen and, in the case of larger pores, egress of effector proteins or whole bacteria into the cytosol. The VAIL system immediately responds to proton leakage and act to diminish the damage. In certain cases, such as during *Salmonella* infection (Ellison et al., 2020; Boyle et al., 2023), asymmetry of lysosomal membrane lipids is also disturbed leading to activation of the STIL system, responding to cytosolic exposure of SM through TECPR1, to yield a versatile CASM response. Interestingly, the STIL response appears to be faster than the exposure of luminal glycans, meaning that asymmetry defects precede the breakage of the membrane (Ellison et al., 2020). The cause of asymmetry deterioration during bacterial infection is not yet clear but may be due to effects on endogenous scramblases by bacterial effectors (Ellison et al., 2020; Barisch et al., 2023).

Another well-documented process is that of influenza A virus (IAV) infection, where a viral protein is involved to generate pores in the membranes of the endolysosomal system (Pinto et al., 1992; Chizhmakov et al., 1996; Durgan and Florey, 2022). During infection, proton conducting channels are formed by the M2 viroprotein, which has multiple functions during the viral life cycle. Proton gradient dissipation leads to activation of VAIL and atg8ylation of affected membranes (Fletcher et al., 2018; Ulferts et al., 2021; Hooper et al., 2022).

Recent findings (Fischer et al., 2020; Liu et al., 2023) have revealed an example that also endogenous pore-forming proteins can be activated and engaged as part of a response to infection through VAIL. The protein STING (Stimulator of interferon genes) classically acts at the transcriptional level to induce expression of interferons and cytokines. In addition, when activated, dimerized STING is transported to endolysosomes and forms proton conducting channels in the membrane which will induce CASM (Liu et al., 2023; Bentley-DeSousa et al., 2025).

### 3.2 Ionophores and lysosomotropic drugs

Many recent experimental studies on lysosomal damaging phenomena utilize substances that in certain aspects mimick those of pathogens, but they also have a value in its own right as such chemicals often are used as drugs in human and veterinary medicine (Breiden and Sandhoff, 2019a). To distinguish damage caused by chemicals taken up by cells from that triggered by viruses and microorganisms, the former is referred to as “sterile” damage. The term “ionophore” is used to describe a set of amphiphilic molecules that bind different cations and partition into bilayers, enhancing ion movement through membranes (Ekinci et al., 2023). In the endolysosomal system, treatments with ionophores are often accompanied with an increase in luminal pH, resulting from an exchange of cations with protons, leading to activation of VAIL through V-ATPase (Florey et al., 2015). The disturbance of ionic balance results in swelling of the compartments due to osmosis that eventually may lead to rupture of the membrane (Jacquin et al., 2017). Monensin and Nigericin are two commonly used ionophores in CASM research. Interestingly, these drugs do not elicit a STIL response, indicating that the asymmetry of lipids is intact, at least for SM (Kaur et al., 2023), and they can therefore be used in experiments to selectively activate the VAIL axis of CASM.

While ionophore drugs affect ionic balances in multiple cellular compartments, lysosomotropic agents predominantly alter conditions within acidic organelles, such as lysosomes (Durgan and Florey, 2022; Meyer and Kravic, 2024). Many lysosomotropic drugs are weak nitrogen bases that possess amphiphilic properties. Upon diffusing into the lysosome, these molecules become protonated at low pH and are therefore trapped, leading to their enrichment in the lumen. Such agents are often referred to as cationic amphiphilic drugs (CADs), which consume protons and partially buffer the lysosomal environment at a higher-than-normal pH. The increase in pH is recognized by the VAIL-system through V-ATPase (Durgan and Florey, 2022). Certain lysosomotropic agents inhibits the activity of luminal acid sphingomyelinase. This enzyme is normally protected from degradation by charge-dependent binding to internal membranes of lysosomes containing the lipid BMP (which is unique among the membrane phospholipids being negatively charged also at lysosomal acidity) (Gallala and Sandhoff, 2011). Acid sphingomyelinase, together with several other lipases, is thought to be released from internal membranes by lysosomotropic substances through competition, and are quickly degraded by cathepsins in the lumen (Breiden and Sandhoff, 2021). Such drugs are therefore also referred to as FIASMAs (functional inhibitors of acid sphingomyelinase) (Kornhuber et al., 2010). The inhibition leads to build-up of SM and other sphingolipids in the lumen that can affect a variety of processes in lysosomes, such as the maintenance of bilayer asymmetry (Breiden and Sandhoff, 2019a). Several drugs of this class activate the STIL system (Kaur et al., 2023), which may be caused by indirect effects through accumulation of sphingolipid metabolites or by direct surfactant action of the drugs on the lipid bilayer (Meyer and Kravic, 2024).

**FIGURE 2:**
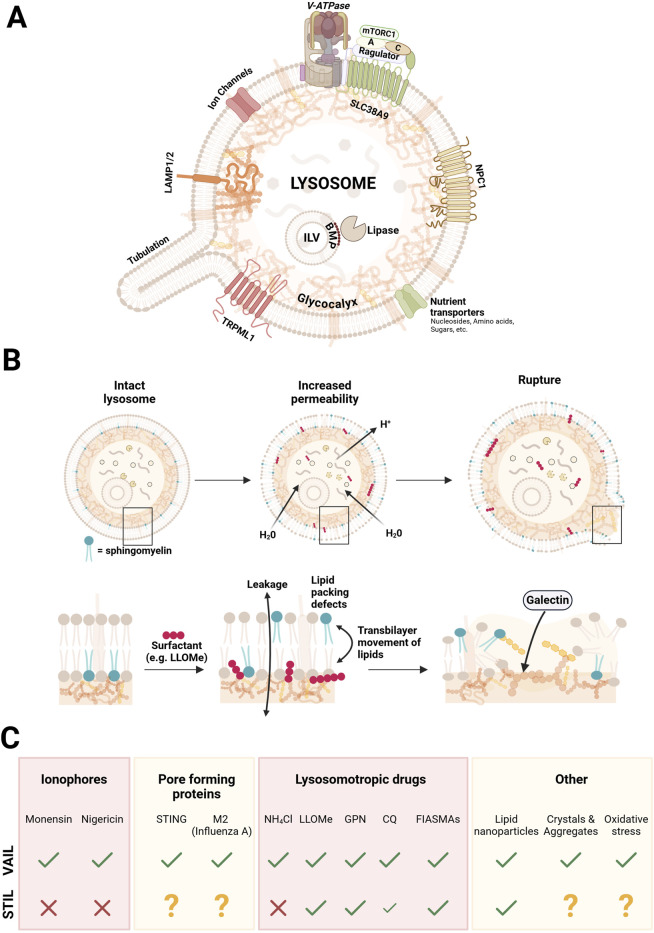
Simplified illustration of the lysosomal structure, stages of surfactant-induced damage, and the CASM response to distinct damage-inducing agents. Panel **(A)** depicts an intact lysosome, highlighting key structural and functional components, including the glycocalyx, intraluminal vesicles (ILVs), ion channels, the major Ca^2+^ channel TRPML1, nutrient transporters, amino acid transporter SLC38A9 with its associated proteins, lysosomal-associated membrane proteins (LAMP1/2), the major cholesterol transporter NPC1, and the V-ATPase complex that maintains the acidic lysosomal environment. Panel **(B)** depicts the progressive stages of lysosomal damage caused by surfactants integrating with the lipid bilayer. The process begins with increased permeability, leading to membrane leakiness and lipid scrambling, and culminates in membrane rupture, marked by exposure of the glycocalyx and recognition by galectins. Panel **(C)** summarizes the response by the two CASM axes (VAIL and STIL) induced by various agents and stressors that cause lysosomal damage, including ionophores, pore forming proteins, lysosomotrophic drugs, lipid nanoparticles, crystals and aggregates, and oxidative stress. A question mark denotes that the effect has not been reported.

Lysosomotropic substances can have a variety of structures and their biological effects are therefore different. The most commonly used lysosome damaging lysosomotropic agent in recent reports is LLOMe (L-leucyl-L-leucine methyl ester). LLOMe is quickly taken up by cells and gets enriched in lysosomes where it is cleaved by cathepsin C, and a mixture of polymers ((leucyl-leucin)_n_-OMe) is generated through dipeptidyl transferase activity of the enzyme (Thiele and Lipsky, 1990; Repnik et al., 2017). The mechanism of action is not entirely clear, but the polyleucine products are thought to act as weak surfactants, affecting the organization of the lysosomal bilayer lipids, leading to transiently increased water and proton permeability (Repnik et al., 2017; Meyer and Kravic, 2024) (Figure 2). A similar drug is the dipeptide GPN (glycyl-L-phenylalanine 2-naphtylamide) (Jadot et al., 1984), but this compound may have effects different from LLOMe in terms of Ca^2+^ permeability and osmosis (Chen et al., 2024). An early effect in cells after administration of LLOMe or GPN is the appearance of SM on the cytosolic side of lysosomes which is detected by SM-reporters, and by TECPR1 inducing STIL (Kaur et al., 2023), indicating that lipid asymmetry is disturbed by these agents (Figure 2).

Weak surfactants are amphiphilic substances that have a propensity to intercalate with membrane lipids and perturb lipid order without solubilizing the membrane (Meyer and Kravic, 2024). Several agents that induce an early STIL response in cells may in fact act as surfactants on the limiting membrane bilayer, as exemplified by LLOMe and certain CADs, which could affect lipid asymmetry (Repnik et al., 2014; Meyer and Kravic, 2024). In line with this, a recent report showed that LLOMe treatment of cultured cells caused rapid lipid-packing defects in the lysosomal limiting membrane that were specifically sensed by an amphipathic helix-containing protein in the cytosol (SPG20) (Gahlot et al., 2024). It may be that the STIL effects that we see with various drugs is due to surfactant action by the drug itself, mediating translocation of SM to the cytosolic side (Meyer and Kravic, 2024). In addition, effects on potential scramblases to disturb asymmetry (several of which are still to be identified) (Niekamp et al., 2022), or the formation of pores in the membrane (Meyer and Kravic, 2024), cannot be ruled out. Indeed, as mentioned above, infection of cells with *Salmonella* induced a similar phenotype of SM exposure on bacteria-containing vacuoles, which may argue for more specific effects on the bilayer (Ellison et al., 2020; Boyle et al., 2023). Ammonium chloride, Chloroquine (CQ), and Hydroxychloroquine (HCQ) are all lysosomotropic compounds that diffuse into lysosomes in their uncharged basic forms and then become protonated, raising the lysosomal pH, thereby inducing VAIL (Jacquin et al., 2017). Because CQ and HCQ can accept two protons, they undergo further protonation and thus accumulate more extensively than the ammonium ion (Chen and Geiger, 2020), which may lead to greater osmotic stress and explain why they—unlike ammonium chloride—can also induce STIL (Wang Y. et al., 2023), possibly through membrane rupture.

Thus, while ionophores selectively trigger VAIL without disrupting SM asymmetry, lysosomotropic agents and FIASMA-type compounds often exhibit broader effects. These include raising lysosomal pH, acting as weak surfactants, or inhibiting key lysosomal enzymes such as acid sphingomyelinase, ultimately culminating in STIL activation. Notably, ammonium chloride serves as an exception among lysosomotropic agents; while it neutralizes lysosomal pH and activates VAIL, it does not induce STIL (Kaur et al., 2023). This distinction is likely due to its monoprotic nature and lack of surfactant activity, which prevent significant perturbation of the lysosomal membrane.

### 3.3 Lipid nanoparticles (LNPs)/vaccines/transfection reagents

One of our early findings, when testing different agents for their effects on TECPR1-dependent CASM, was the surprising result that certain common transfection reagents, such as JetMessenger®, gave a strong STIL response (Kaur et al., 2023). This indicated that the formulation affects the limiting membrane, leading to exposure of SM, which may be one of the reasons for the enhancement of polynucleotide delivery. The same result was obtained with clinically relevant lipid nanoparticles (DLin-MC3-DMA), initially used for delivery of siRNA and lately also for mRNA vaccines (Karp and Peer, 2018; Akinc et al., 2019). To enhance the endosomal escape of polynucleotides, modern formulations of LNPs, such as those used in mRNA vaccines, consist of a mixture of four different lipid constituents: a phospholipid, an ionizable lipid, cholesterol, and a PEG (polyethyleneglycol)-modified lipid (Dowdy, 2017; Mukai et al., 2022; Wu et al., 2024). The effect in lysosomes is mediated by the ionizable lipid, having a role similar to the lysosomotropic drugs described above, affecting the organization of the limiting membrane and permitting escape of its cargo through the membrane. The cellular response is immediate, orchestrating a powerful CASM reaction through recognition of SM exposure in addition to activation of VAIL (Kaur et al., 2023). It can be envisioned that elaboration with the CASM response in future research may have a potential to enhance the efficiency of vaccine delivery.

### 3.4 Other types of processes that can elicit a CASM response

The generation, or uptake into cells, of substances that have a propensity to form crystals or large aggregates have also been shown to provoke a CASM response. Examples are silica, ureate, and cholesterol crystals, and amyloid proteins such as α-synuclein, amyloid-β, and tau (Papadopoulos and Meyer, 2017; Papadopoulos et al., 2020; Meyer and Kravic, 2024). A current view is that the accumulation of large structures in lysosomes will invoke penetration of the limiting membrane leading to leakage of ions and enzymes into the cytosol. The VAIL system is activated (Durgan and Florey, 2022), but since the damage often is severe the main response appears to rely on macrolysophagy (through exposure of luminal glycans and ubiquitination) to remove the damaged lysosomes (Maejima et al., 2013).

An alternative CASM-dependent mechanism associated with aggregated proteins is LANDO (LC3-associated endocytosis), which was identified in a murine model of Alzheimer’s disease (Heckmann et al., 2019). In this pathway, Rab5-positive endosomes become decorated with ATG8 in response to amyloid-β build-up, ultimately triggering innate immune responses and inflammation. However, because LANDO takes place on early endosomal compartments and has been reviewed elsewhere (Magne and Green, 2022; Pena-Martinez et al., 2022), we will not address it further here.

Oxidative stress on cells can alter the lipid metabolism in lysosomes resulting in products, such as peroxidated lipids, that may have harmful consequences on lysosome function due to effects on its limiting membrane, causing, for example, lipid-packing defects (Gahlot et al., 2024; Meyer and Kravic, 2024). Also physiological processes may create reactive oxygen species (ROS) that is recognized by CASM. The best example is that of LAP (LC3-associated phagocytosis), which actually was the first described case of “non-canonical autophagy” (Sanjuan et al., 2007). In LAP, phagocytosis of pathogens or dead cells leads to formation of phagosomes, which matures into degradative compartments through a series of reactions culminating in the production of ROS by the enzyme NOX2 (NADPH oxidase 2). This reaction consumes protons that is sensed by the VAIL system and results in atg8ylation of the phagosome membrane (Hooper et al., 2022).

## 4 Downstream effectors of CASM

**FIGURE 3:**
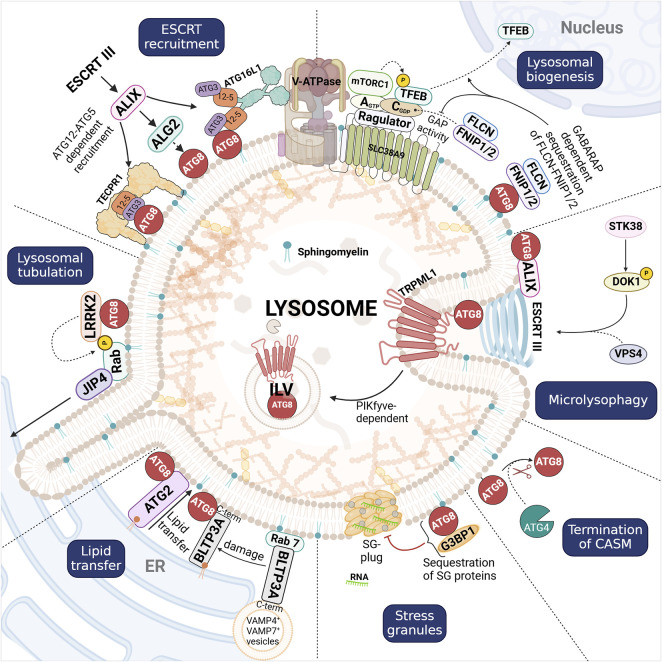
Overview of CASM pathways activated in response to lysosomal damage, and the role of membrane-anchored ATG8 proteins (red filled circles), as detailed in the main text. CASM supports Lysosomal biogenesis through TFEB activation, where the inhibition of FLCN-FNIP1/2 via GABARAP enables TFEB nuclear translocation to drive lysosomal gene expression and stress response pathways. ESCRT recruitment is initiated by the recruitment of ALIX and ESCRT-III components to ATG12-ATG5/GABARAP on damaged lysosomal membranes, facilitating membrane scission and resealing. Lysosomal tubulation is driven by the CASM-dependent recruitment of LRRK2, which phosphorylates Rab GTPases to promote tubule formation and membrane sorting. Lipid transfer through BLTPs (BLTP3A and ATG2) at ER-lysosome contact sites, provide essential lipids to restore and stabilize membrane integrity. Stress granule formation regulated by ATG8, which sequesters components like G3BP1 to prevent granule assembly. Termination of CASM occurs through ATG4-dependent cleavage of lipidated ATG8. Microlysophagy involves CASM-induced PIKfyve-dependent intraluminal vesicle (ILV) formation, which contributes to membrane turnover and damage resolution. Although CASM can proceed via two branches (VAIL and STIL), it remains unclear whether either pathway yields distinct downstream effects; for this reason, we have not distinguished between VAIL- and STIL-mediated ATG8 conjugation in the figure.

Since the initial characterization of non-canonical autophagy, or CASM, a key question has been to elucidate the role of ATG8 proteins downstream of their membrane attachment. It has become evident that the ATG8 conjugation machinery is adept at detecting membrane damage or insults, and that ATG8 proteins, acting as a scaffold, together with specific cellular factors like phosphoinositides and Rab GTPases, facilitate the recruitment and stabilization of various protein complexes required for lysosomal repair and maintenance. This relies on effector proteins distinct from those involved in canonical autophagy, emphasizing the role of additional factors in determining specificity of ATG8 interactions. Here, we explore key effector proteins recruited by CASM and their roles in addressing lysosomal damage (Figure 3).

### 4.1 Lysosomal tubulation events

Lysosomal tubules are dynamic membrane structures that form from lysosomes and related organelles under various physiological and pathological conditions—such as shifts in nutrient availability, cellular stress, and organelle damage—thereby helping to maintain lysosomal homeostasis and participating in essential recycling processes like autophagic lysosome reformation (ALR) and phagolysosome resolution (Yu et al., 2010; Krajcovic et al., 2013).

During ALR, which follows autophagy, mTOR reactivation at autolysosomes triggers the formation of tubular extensions. These tubules then undergo scission to produce proto-lysosomes that mature into fully functional lysosomes (Yu et al., 2010). A comparable mechanism operates in antigen-presenting cells, where lysosomal tubules form from phagosomes through phagolysosome resolution, contributing to antigen processing and presentation (Krajcovic et al., 2013; Lancaster et al., 2021). Lysosome reformation can also occur from endosomal compartments, where hybrid endosome-lysosome structures are generated. These structures undergo fission and maturation to regenerate lysosomes, thereby supporting lysosomal homeostasis and recycling under specific physiological conditions (Pryor et al., 2000; Bright et al., 2005).

However, when lysosomes are damaged, a CASM-dependent pathway produces an unconventional class of tubules lacking the lysosomal membrane protein LAMP-1. A principal driver of this CASM-dependent tubulation is Leucine-Rich Repeat Kinase 2 (LRRK2) (Eguchi et al., 2024; Bentley-DeSousa et al., 2025).

LRRK2 is a large, multifunctional kinase that phosphorylates members of the Rab GTPase family, such as Rab8A, Rab10, and Rab35 (Steger et al., 2016; Steger et al., 2017). It is broadly expressed throughout the body, and its dysfunction has been associated with Parkinson’s Disease (PD) and Crohn’s Disease (CD) (Hui et al., 2018). *In vivo*, loss or inhibition of LRRK2 leads to enlarged lysosomes in organs like the kidney and lung, while familial LRRK2 mutations alter lysosomal morphology (Henry et al., 2015; Hockey et al., 2015).

Under lysosomal stress, LRRK2 is recruited to lysosomes through a CASM-dependent mechanism by directly interacting with GABARAP via two LIR motifs (Bentley-DeSousa et al., 2025), a process triggered by diverse stressors—such as Chloroquine, Monensin, Nigericin, STING agonists, TRPML1 agonists, and LLOMe—and also occurring during LC3-associated phagocytosis (LAP) (Eguchi et al., 2024; Bentley-DeSousa et al., 2025). Importantly, CASM is essential for LRRK2 recruitment; depletion of CASM disrupts LRRK2 localization, whereas knockdown of autophagy-essential genes like ULK kinase complex subunits FIP200 or ATG13 does not, highlighting a CASM-specific, rather than a general atg8ylation-dependent pathway (Eguchi et al., 2024). Bentley-DeSousa et al. (2025) further proposed that LRRK2’s preference for CASM-positive compartments may be explained by a coincidence detection mechanism that relies on the co-recognition of GABARAP and specific Rab GTPases (e.g., Rab10, Rab12, Rab29, Rab32), which collectively facilitate its recruitment (Bentley-DeSousa et al., 2025).

Once at compromised lysosomes, LRRK2 phosphorylates Rab GTPases, inhibiting their interaction with GDP dissociation inhibitors (GDIs) and thus keeping them active on the lysosomal membrane (Bonet-Ponce et al., 2020). Activated Rabs recruit JNK-interacting protein 4 (JIP4) to lysosomal surfaces, with Rab10 playing the primary role. JIP4 then facilitates the formation of LRRK2-driven tubules, known as LYTL (LYsosomal Tubulation/sorting driven by LRRK2) (Bonet-Ponce et al., 2020). These tubules, which lack LAMP-1/LIMP-2 and Dextran-555 labeling, differ from ALR tubules both in composition and in their utilization of distinct motor proteins, underscoring separate mechanistic pathways (Bonet-Ponce et al., 2020).

The pathogenic G2019S mutation in LRRK2, associated with PD, enhances its recruitment to compromised lysosomes, leading to excessive JIP4 recruitment and lysosomal tubulation (Bonet-Ponce et al., 2020). This excessive remodeling of lysosomal membranes underscores the central role of LRRK2 in lysosomal dynamics and its broader implications in disease mechanisms. While the specific roles of the LRRK2-driven tubules are not yet fully understood, they are unlikely to participate in proto-lysosome reformation, as they lack lysosomal membrane markers such as LAMP-1 and LIMP-2.

In contrast, a distinct lysosomal tubulation pathway mediated by TBC1D15—a Rab7 GTPase-activating protein (GAP)—also relies on atg8ylation at lysosomes yet independently of a “classical” CASM mechanism (Bhattacharya et al., 2023). Following acute lysosomal damage, TBC1D15 is recruited to damaged membranes through interactions with ATG8 proteins. Acting as a scaffold, TBC1D15 assembles and stabilizes components of the lysosomal tubulation machinery—including dynamin-2, kinesin-5B, and clathrin—thereby driving tubule formation and scission to promote lysosomal recovery independently of lysosomal biogenesis. Surprisingly, TBC1D15 recruitment and the resulting tubulation is not dependent on VAIL, as normal TBC1D15 recruitment is seen with a CASM-deficient ATG16L1 mutant. Instead, it seems that TBC1D15 relies on canonical macroautophagy factors (e.g., ATG13) for its recruitment and function in lysosomal tubulation and recovery.

### 4.2 Lysosomal biogenesis

Transcription factor EB (TFEB) serves as a master regulator of the autophagy-lysosomal pathway by driving gene expression through its binding to Coordinated Lysosomal Expression and Regulation (CLEAR) elements in target gene promoters (Sardiello et al., 2009). Its activity is tightly regulated by mTORC1, which phosphorylates TFEB to retain it in the cytosol, preventing its nuclear translocation (Martina et al., 2012; Roczniak-Ferguson et al., 2012; Settembre et al., 2012). However, accumulating evidence now indicates that CASM can override this block on TFEB, even in contexts where mTORC1 remains active toward other targets.

Unlike other mTORC1 targets, such as S6K and 4EBP1, TFEB requires active RagC to be recruited to the lysosome for phosphorylation (Napolitano et al., 2020). RagC’s activation, converting it to its GDP-bound state, is mediated by the GAP complex FLCN-FNIP1/2 (Tsun et al., 2013).

CASM supports TFEB’s nuclear translocation by utilizing a GABARAP-specific LIR motif within FNIP1 and FNIP2 (Goodwin et al., 2021). Lipidated GABARAP binds this motif, sequestering the FLCN-FNIP1/2 complex and preventing it from activating RagC. As a result, RagC remains inactive, allowing TFEB to dissociate from the lysosome and move into the nucleus (Goodwin et al., 2021).

Several agents can trigger CASM-mediated TFEB translocation, including STING agonists, TRPML1 agonists, and the ionophore Monensin (Nakamura et al., 2020; Goodwin et al., 2021; Lv et al., 2024). The GABARAP-dependent sequestration of FLCN-FNIP1/2 has also been observed in xenophagy and mitophagy, indicating that this mechanism extends beyond CASM (Goodwin et al., 2021; Schmuckli-Maurer et al., 2024). Interestingly, high concentrations of STING agonists can induce TFEB translocation through CASM-independent pathways, suggesting the presence of additional regulatory layers that remain to be explored (Lv et al., 2024).

### 4.3 Lipid transfer proteins

Maintaining the lysosomal membrane is crucial for protecting the cytoplasm from the harsh lysosomal environment. When lysosomes are damaged or permeabilized, lipids are rapidly introduced to restore and protect membrane integrity. The endoplasmic reticulum (ER) serves as the main source of these lipids, and upon lysosomal injury, specialized ER–lysosome membrane contact sites (EL-MCS) form to facilitate non-vesicular lipid transfer (Radulovic et al., 2022; Tan and Finkel, 2022; Hanna et al., 2023).

A key factor in this process is PI(4)P on lysosomal membranes, generated by PI4K2A (Radulovic et al., 2022; Tan and Finkel, 2022). PI(4)P drives the formation of EL-MCS and helps recruit oxysterol-binding protein (OSBP) and oxysterol-binding protein-related proteins (ORPs). These shuttle-like proteins bind lipids in a hydrophobic cleft, extracting them from the ER and delivering them to lysosomes across an aqueous gap (Radulovic et al., 2022; Tan and Finkel, 2022). For instance, ORP1L transfers cholesterol in exchange for PI(4)P, whereas ORP9, ORP10, and ORP11 transport PS. Notably, PS on lysosomal membranes activates ATG2, a bridge-like lipid transfer protein (BLTP) capable of bulk lipid transport (Tan and Finkel, 2022).

BLTPs, such as ATG2 (BLTP4), adopt a rod-like structure with a hydrophobic groove optimized for lipid transfer (Hanna et al., 2023). They feature a repeating β-groove (RBG) domain, whose role in lipid selectivity remains unclear, and a chorein motif in the N-terminal region, typically localized to the ER which is the lipid donor (Leonzino et al., 2021; Hanna et al., 2023). Structural studies reveal that BLTPs can accommodate a single lipid at the groove’s narrowest point, supporting unidirectional lipid flow. These proteins bridge membrane gaps at contact sites, enabling low-energy bulk lipid transfer essential for membrane repair (Hanna et al., 2023). This mechanism highlights their role in preventing further membrane damage through efficient lipid replenishment.

Recent work shows that BLTPs can interact with ATG8 on lysosomes in a CASM-dependent manner. For example, ATG2 and ATG8 associate on lysosomal membranes after permeabilization and osmotic stress (Cross et al., 2023). While this interaction is not strictly required for ATG2’s recruitment, it helps stabilize ATG2 at the damaged lysosome. BLTP3A, in contrast, is recruited to lysosomes directly through CASM (Hanna et al., 2025). Under normal conditions, BLTP3A localizes to VAMP7- and VAMP4-positive vesicles tethered via its N-terminal region to lysosome-bound Rab7. Upon damage, Rab7-dependent tethering is disrupted, but CASM leads to BLTP3A reassociation through a LIR domain in its C-terminal region. This interaction appears to expose the protein’s N-terminal chorein motif, enabling ER engagement and lipid transfer to the lysosome.

Because BLTPs only access the cytosolic leaflets at MCS, lipid flow can unbalance the bilayer of donor and acceptor membranes (Hanna et al., 2023). Therefore, BLTPs cooperate with scramblases for balancing this asymmetry during lipid flow at MCS. For example, *in vitro* work has demonstrated that ATG2 can interact with scramblases on both donor membranes (VMP1 and TMEM41B) and acceptor membranes (ATG9) in an artificial system modeling autophagosome biogenesis (Ghanbarpour et al., 2021). However, a model explaining how BLTP-induced leaflet imbalance is managed following lysosomal damage has yet to be proposed.

In addition to ATG2 and BLTP3A, VPS13C (also known as BLTP5C) has been observed on lysosomes following damage, apparently independent of VAIL; whether its recruitment depends on STIL remains to be tested. As a BLTP, VPS13C is expected to promote net lipid flow to compromised membranes (Cai et al., 2022; Wang et al., 2025).

Increasing evidence suggests that CASM is vital for stabilizing BLTPs (e.g., ATG2 and BLTP3A) at lysosomes, though the exact molecular details remain unclear. This mechanism may enable CASM to compensate for membrane thinning and prevent rupture. Although several studies have examined lipid transfer following membrane-perforation damage, the impact on de-acidified and swelling lysosomes—where VAIL is triggered but the limiting membrane remains intact—remains largely unexplored.

### 4.4 ESCRT recruitment

The Endosomal Sorting Complexes Required for Transport (ESCRT) machinery is essential for a variety of cellular processes, including multivesicular endosome formation, virus budding, cytokinetic abscission, and nuclear envelope reassembly (Christ et al., 2017; Vietri et al., 2020). It also plays a crucial role in membrane repair, including restoring integrity of the plasma membrane and lysosomes following damage. The ESCRT machinery consists of four subcomplexes: ESCRT-0, ESCRT-I, ESCRT-II, and ESCRT-III. ESCRT-I is crucial for recruiting ESCRT-III, with ALG2-interacting protein X (ALIX), a Bro1 domain-containing protein, serving as a bridge between them (Christ et al., 2017).

As demonstrated by Skowyra et al. (2018) and Radulovic et al. (2018), the reparative role of ESCRTs in lysosomal membrane damage precedes macrolysophagy, with TSG101 (an ESCRT-I component) and ALIX (a critical ESCRT-nucleating factor) being recruited to damaged lysosomes before the appearance of galectin-3, which marks severe membrane damage (Radulovic et al., 2018; Skowyra et al., 2018). This early recruitment is considered as the first line of defense against membrane damage, as failure in this repair mechanism can lead to irreversible damage and cell death. In addition, ESCRTs recruitment to damaged lysosomes is dependent on Ca^2+^, where cytoplasmic Ca^2+^ signals the recruitment of ALG-2, which, upon binding to the membrane, undergoes a conformational change and interacts with ALIX (Skowyra et al., 2018; Shukla et al., 2022; Chen et al., 2024). Besides repairing membrane perforations caused by LLOMe, ESCRTs are activated by elevated peri-lysosomal Ca^2+^ levels, which can result from TRPML1 activation even without membrane perforation, thereby protecting lysosomes from rupture (Chen et al., 2024).

It has also been reported that ALIX recruitment occurs in a GABARAP-dependent manner during microlysophagy (discussed below) triggered by lysosomal damage or osmotic stress (Ogura et al., 2023). In this study, cells lacking the atg8ylation machinery (ATG16L1, ATG7, ATG8 or ATG3) failed to recruit ESCRT components (ALIX, VPS4 and CHMP4B) following lysosome damage. In contrast, autophagy deficient FIP200 knockout (KO) cells showed no defect in ESCRT recruitment. Further analysis revealed that ALIX directly interacts with ATG8 proteins, with specificity for the GABARAP subfamily. This finding highlighted the essential role of CASM in mediating ALIX dependent nucleation of the ESCRT machinery on compromised lysosomes (Ogura et al., 2023).

Later, another study found that ALIX recruitment following membrane damage is primarily dependent on ATG12-ATG5, which is recruited by either ATG16L1 or TECPR1, rather than on GABARAP (Corkery et al., 2024). This study showed that ALIX could still be recruited in the absence of atg8ylation, provided ATG12-ATG5 was present. However, in atg8ylation deficient cells, the ESCRT machinery’s distribution on damaged membranes was fragmented and incomplete. This impaired recruitment was attributed to the lack of CASM-dependent stabilization of ALG-2 on damaged lysosomes, facilitated by a direct interaction with LC3B. Importantly, lysosome recovery, following damage, was equally impaired in ATG8 KO cells as it was in cells lacking ATG5, or ATG16L1 and TECPR1 (Corkery et al., 2024), highlighting the necessity of both the ATG8 conjugation machinery and CASM for efficient ESCRT-mediated repair (Corkery et al., 2024).

An interesting phenomenon to note is the formation of an alternative ATG12 complex, where ATG12 is conjugated to ATG3 instead of ATG5, a situation that becomes more pronounced with ATG5 depletion (Murrow et al., 2015; Wang F. et al., 2023). This ATG12-ATG3 complex, which lacks the ability to interact with ATG16L1 or TECPR1, has been shown to impact ALIX-dependent pathways, such as exosome biogenesis and lysosomal exocytosis. In the absence of ATG5, ALIX exhibits a strong affinity for ATG12-ATG3, impairing its recruitment to lysosomes and compromising the lysosomal repair process.

Together, these studies underline the vital role of ESCRTs as a first line of defense against lysosomal damage and highlight the intricate contributions of CASM in optimizing ESCRT function. While ESCRT components such as TSG101 and ALIX initiate repair in a Ca^2+^-dependent manner, the atg8ylation machinery ensures the proper stabilization and distribution of ESCRT proteins on compromised membranes. Moreover, the interplay between distinct E3-like ligase complexes (ATG16L1–ATG12–ATG5 vs. TECPR1–ATG12–ATG5) and the alternative formation of ATG12–ATG3 reveal additional layers of complexity. These findings emphasize the critical interplay between CASM and ESCRTs in lysosomal repair, with CASM not only stabilizing key repair components but also dictating the spatial organization of ESCRT machinery required for effective membrane restoration.

### 4.5 Microlysophagy

Microautophagy is a selective process that removes organelles and cytoplasmic components through invagination of the lysosomal limiting membrane, a mechanism observed in both yeast and mammalian systems (Sahu et al., 2011; Schuck et al., 2014). Various cargoes—including mitochondria, ER, peroxisomes, and nuclear material—have been described to undergo selective microautophagy. A specialized form of this pathway, known as microlysophagy, mediates lysosome membrane turnover in mammalian cells independently of macroautophagy, facilitating lysosome size control through the formation of ILVs. Microlysophagy can be divided into ATG8-dependent and ATG8-independent processes, both relying on the ESCRT machinery, with the ATG8-independent route rather requiring ubiquitin (Wang L. et al., 2023).

ATG8-dependent microlysophagy requires the core conjugation machinery (ATG5, ATG7, and ATG3), but it does not depend on the ULK-complex (ATG13 and ULK1) required for macroautophagy (Lee et al., 2020). Under conditions of extreme cellular stress, lysosomal turnover becomes essential for reducing lysosomal damage. For instance, stressors such as LLOMe or ionophores induce CASM on lysosomes, and trigger the degradation of select lysosomal membrane proteins, including TRPML1 and SNAT7 (Lee et al., 2020). In earlier sections, we discussed how CASM is vital for recruiting the ESCRT machinery that mediates ILV formation in response to agents like Monensin and LLOMe. Recent findings, however, have highlighted additional factors involved in the ATG8-dependent microlysophagy pathway.

Specifically, both the phosphoinositide kinase PIKfyve and the lysosomal Ca^2+^ channel TRPML1 act as critical downstream effectors in CASM-dependent microlysophagy. Monensin-induced ILV formation is disrupted when either PIKfyve or TRPML1 is inhibited (Klein et al., 2024). Notably, blocking either protein does not reduce Monensin-induced LC3 lipidation, rather, it appears to enhance it, indicating that TRPML1 and PIKfyve are required for ILV formation but not for LC3 lipidation in ionophore-treated cells. Collectively, these observations underscore the roles of PIKfyve and TRPML1 in lysosomal homeostasis and highlight their importance in orchestrating ILV formation during microlysophagy.

Another study highlighted the essential role of serine-threonine kinase 38 (STK38) in microlysophagy, more specifically a role in the disassembly of the ESCRT complex (Ogura et al., 2023). STK38 achieves this by phosphorylating the scaffold protein DOK1 at a specific serine residue (S269), which facilitates the recruitment of VPS4 to damaged lysosomes. This recruitment is critical for the final disassembly of the ESCRT complex, ultimately leading to the formation of ILVs within lysosomes.

As mentioned above, ESCRT-mediated microautophagy can occur with or without the involvement of CASM (Li et al., 2015; Ogura et al., 2023). However, the factors determining which pathway is prioritized remain unclear. Under conditions of severe lysosomal stress, it is plausible that membrane atg8ylation accelerates ESCRT-mediated microlysophagy. Additionally, the specific circumstances under which ESCRT- and PIKfyve-dependent microlysophagy are preferentially activated require further investigation, highlighting the intricacy of lysosomal quality control and size regulation.

### 4.6 Stress granule assembly at lysosomal damage sites

We will also briefly mention stress granules (SGs), as atg8ylation and potentially CASM have been implicated in their formation. SG are cytoplasmic, membrane-less liquid-liquid phase-separated biomolecular condensates that contain translation factors, mRNAs, RNA-binding proteins (RBPs), including G3BP1, and other associated proteins, such as NUFIP2.

The formation of SGs can be induced by lysosomal damage (Jia et al., 2022). Interestingly, *in vitro* experiments using giant unilamellar vesicles (GUVs) have shown that G3BP1-RNA condensates can stabilize damaged membranes and prevent the leakage of luminal content, while their absence results in vesicle collapse (Bussi et al., 2023). Consistently, the inhibition of SG formation (via knockdown of G3BP proteins) affects ESCRT-mediated repair and pushes damaged lysosomes towards macrolysophagy (Bussi et al., 2023).

SG formation increases in situations where the CASM machinery is compromised (Jia et al., 2022). Additionally, it was demonstrated that GABARAP, when conjugated to the lysosome, suppresses SG formation by sequestering essential components G3BP1 and NUFIP2 through direct interaction (Jia et al., 2022).

### 4.7 Termination of lysosome-associated CASM

A key hallmark of CASM, as mentioned above, is the conjugation of ATG8 proteins not only to PE but also to PS (Durgan et al., 2021). This has been observed across a range of CASM-inducing stimuli, including pharmacological treatments with Monensin or LLOMe, LC3-associated phagocytosis, and influenza A virus infection (Durgan et al., 2021; Cross et al., 2023).

In both autophagy and CASM, the terminal step for ATG8 proteins involves either their degradation or de-lipidation. De-lipidation in humans is carried out by the ATG4 protease family, which consists of four isoforms: ATG4A, ATG4B, ATG4C, and ATG4D (Scherz-Shouval et al., 2003; Tanida et al., 2004; Kauffman et al., 2018). Notably, ATG4D exhibits a unique role in CASM, as it demonstrates little to no activity toward ATG8-PE but shows the highest activity among ATG4 isoforms toward ATG8-PS (Durgan et al., 2021).

This specificity of ATG4D for ATG8-PS, rather than ATG8-PE, elucidates a key mechanism for distinguishing CASM from autophagy. It raises two important considerations: (1) ATG8-PS may recruit different interaction partners, and (2) ATG4 isoforms may selectively target ATG8 attached to different lipids to regulate its removal during autophagy versus CASM. An alternative explanation suggests that conjugating ATG8 to PS could alter the lipid’s biophysical properties, thereby influencing charge-dependent processes, for example, during phagocytosis (Yeung et al., 2009; Durgan et al., 2021).

## 5 Concluding remarks

From the research done on CASM so far, it is clear that ATG8 decoration of damaged lysosomal membranes can occur through different mechanisms, and also that the outcome can vary depending on cell-type, the nature of the damage, and the duration of the insult. As new data emerge, one can start to see certain patterns in how cells respond to lysosomal injury in ways that rely on, or are enhanced by, ATG8. Nevertheless, many outstanding questions remain before we can fully appreciate the integrated system in which CASM operates, and its precise roles in different cellular contexts continue to be an active area of investigation.

In several cases it appears that atg8ylation on single membranes acts to stabilize the membrane and to provide efficient platforms for recruitment of key proteins, perhaps to enhance the kinetics of repair (Radulovic et al., 2018; Skowyra et al., 2018), removal (Lee et al., 2020), or formation of new membrane (Cross et al., 2023; Eguchi et al., 2024; Bentley-DeSousa et al., 2025). Thus far, almost all studies report that ATG8-dependent recruitment of factors occur via interactions between LIR-sequences in the recruited proteins and the LIR-docking site on membrane-anchored ATG8s. Minor variations on this theme has been described (Johansen and Lamark, 2020), but in essence it seems that an early response after damage is to present anchor points for dedicated proteins involved in various specific processes as outlined above. Mechanistically, although it may be possible to implement, for example, a repair process without ATG8s, the kinetics is likely enhanced several-fold if factors are enriched at certain sites. This principle may be especially important when the cell has to deal with potentially life-threatening danger.

Generally, the effects of lysosomal damage can be divided into early and late phases. Early on, events such as Ca^2+^-leakage through the Ca^2+^-specific channels (Hu et al., 2024; Meyer and Kravic, 2024), loss of proton gradient (Durgan and Florey, 2022; Figueras-Novoa et al., 2024), disruption in membrane lipid-packing (Gahlot et al., 2024), and collapse of membrane leaflet asymmetry (leading to SM exposure) take place (Ellison et al., 2020; Niekamp et al., 2022). These cues trigger ATG8 lipidation via VAIL or STIL, enabling repair mechanisms like microlysophagy, where CASM facilitates the internalization of small damaged membrane portions to stabilize and restore lysosomal function. If the damaging agent is removed at this stage, the cell can heal the membrane. At a late stage, however, severe breakdown exposes internal glycans in the glycocalyx (Gahlot et al., 2024; Jacob and Gorek, 2024), rendering salvage futile as much of the lysosomal contents escape. The irreversibly damaged lysosome is then cleared through macrolysophagy, dependent on galectins and ubiquitination, with autophagy receptors like TAX1BP1 linking ubiquitin-tagged lysosomes to ATG8-decorated phagophores (Eapen et al., 2021). One intriguing possibility is that the early CASM at the lysosomal membranes might also serve to enhance a subsequent macrolysophagy response, should it be needed, and thereby reduce the risk of excessive leakage of lysosomal contents. Still, CASM’s involvement in macrolysophagy is uncertain, and how it might prepare lysosomes for clearance remains to be explored.

Emerging evidence suggests that, while LC3B-lipidation is often used as a convenient readout in CASM studies, members of the GABARAP subfamily may play a more prominent role in recruiting LIR-containing proteins in both canonical autophagy and CASM (Johansen and Lamark, 2020; Mizushima, 2020). Although much research has focused on direct interactions between ATG8 proteins and LIR motifs, other factors, such as membrane lipid composition (e.g., phosphoinositides), membrane curvature, the type of lipid conjugated to ATG8 (PE or PS), and membrane-associated proteins like Rabs, can substantially influence these interactions. Investigating how these factors act in concert could provide deeper insights into the underlying mechanisms of CASM.

We now know of two distinct systems that drive ATG8-lipidation at lysosomal membranes upon damage, named VAIL and STIL, and more pathways may be uncovered in the future (Deretic, 2024; Deretic and Klionsky, 2024; Figueras-Novoa et al., 2024). VAIL and STIL use different E3-like ligase complexes (ATG16L1-ATG12-ATG5 and TECPR1-ATG12-ATG5, respectively), but otherwise share a similar conjugation mechanism. Their activation, however, differs: VAIL responds to signals from the V-ATPase (Xu et al., 2019), whereas STIL is triggered by SM exposure following membrane asymmetry defects (Boyle et al., 2023; Corkery et al., 2023; Kaur et al., 2023; Wang Y. et al., 2023). This division of labor presumably provides spatio-temporal flexibility in atg8ylation during damage responses, allowing scenarios in which one E3 can act more rapidly, or exclusively, compared to the other. Another interesting difference is that STIL remains unaffected by the bacterial effector SopF, which irreversibly blocks V-ATPase-mediated signaling (Kaur et al., 2023). Thus, during *Salmonella* infections, SopF would selectively inhibit VAIL but leave STIL functionality intact, providing an additional layer of importance to their diverging functionality (Xu et al., 2019; Kaur et al., 2023).

Mouse models deficient in VAIL or STIL have been generated separately, and both show normal macroautophagy (Ogawa et al., 2011; Rai et al., 2019). Interestingly, VAIL-deficient mice are extremely vulnerable to infections with low-pathogenicity IAV, exhibiting high viral replication in the lungs, dysregulated cytokine responses, and lethality rates that mirror those seen with highly pathogenic IAV (Wang et al., 2021). Although TECPR1-deficient mice have yet to be investigated specifically in the context of CASM, TECPR1-deficient MEFs display diminished LC3 responses to infections with *Shigella* ΔicsB, resulting in markedly higher bacterial survival (Ogawa et al., 2011). These observations underscore the importance of disrupting both STIL and VAIL in future research to determine their interplay and to establish how each pathway can compensate for the other in defending against infections.

It also remains to be determined whether STIL alone can be triggered under specific conditions that go beyond SopF-mediated disruptions of VAIL. Pinpointing situations where STIL operates independently would yield insight into its specialized functions in cellular processes and how it contributes to maintaining endolysosomal balance. Moreover, studying mice that lack both STIL and VAIL will be a crucial step toward revealing how these pathways coordinate and back up one another. Observing how double-deficient mice respond to different bacterial and viral infections, as well as to challenges within their endolysosomal networks, could highlight new ways these mechanisms control host defense and cellular stress responses. Such investigations might even uncover new weaknesses that could be targeted for treatments in diseases involving disruptions of these protective pathways.
